# Exploring the Pharmacokinetics and Gut Microbiota Modulation of Hesperidin and Nobiletin from Mandarin Orange Peel in Experimental Dogs: A Pilot Study

**DOI:** 10.3390/metabo15010003

**Published:** 2024-12-25

**Authors:** Jun Nakahigashi, Makoto Kurikami, Satomi Iwai, Sadahiko Iwamoto, Shou Kobayashi, Eiji Kobayashi

**Affiliations:** 1 Agriculture & Foods Research Center, AIR WATER INC., 1-7, Tsukisamu Higashi 2-jo 16-chome, Toyohira-ku, Sapporo 062-0052, Hokkaido, Japan; nakahigashi-jun@awi.co.jp (J.N.);; 2 School of Veterinary Medicine, Kitasato University, 35-1, Higashi 23, Towada 034-8628, Aomori, Japan; 3 Division of Cardiovascular and Genetic Research, Center for Molecular Medicine, Jichi Medical University, 3311-1 Yakushiji, Shimotsuke-shi 329-0498, Tochigi, Japan; 4 Kobayashi Regenerative Research Institute, LLC, 1 Chayano-cho, Wakayama-shi 640-8263, Wakayama, Japan

**Keywords:** mandarin orange peel, flavonoids, hesperidin, Alzheimer’s disease, canine health, gut microbiota, pet nutrition, nobiletin

## Abstract

**Background/Objectives:** Mandarin orange peel (MOP) is recognized for its traditional medicinal properties due to its high flavonoid content. This study aimed to analyze MOP harvested in Japan for specific bioactive compounds and to explore its health applications in dogs, including effects on gut microbiota and cognitive symptoms. **Methods:** Flavonoid content (particularly hesperidin and nobiletin) of MOP was measured. High-flavonoid MOP was then incorporated into gelatin cubes. In the time–course blood concentration study, experimental beagle dogs received a single oral dose (4 g). For intestinal microbiota analysis and blood biochemical tests, beagle dogs were administered 1 g twice daily. For the Demonstration Test, older dogs (a 14-year-old female Shiba Inu, a 14-year-old female Miniature Dachshund, and a 19-year-old male Miniature Dachshund) were administered 1 g twice daily. Analysis included microbiota profiling via 16S rDNA sequencing and observational assessment of cognitive indicators in a pilot study involving senior dogs with Alzheimer’s disease. **Results:** The MOP powder contained 9.3% hesperidin in early-ripening varieties (October harvest) and 6.9% in ripe varieties (December harvest). Nobiletin content was 41 mg/100 g (0.041%) and 35 mg/100 g (0.035%) for the early and late harvests, respectively. Administration of MOP-enriched gelatin cubes reduced *Fusobacteriaceae* and increased *Eggerthellaceae*. Cognitive symptoms like howling and counterclockwise turning showed improvement in senior dogs (*n* = 3). **Conclusions:** This study provides preliminary support for the potential health benefits of MOP in canine dietary applications, particularly for gut health and cognitive function. Improvement in cognitive symptoms may be due to the anxiolytic effects of mandarin peel.

## 1. Introduction

Flavonoids are widely recognized for their antioxidant and anti-inflammatory properties and have been reported to be effective in the prevention and management of various diseases [1,2,3,4,5]. Foods and fruits rich in flavonoids have garnered significant attention as health-promoting options, with citrus fruits particularly noted for their long-documented ability to regulate vascular permeability [6]. Among seasonal fruits, mandarin orange peel (MOP), traditionally introduced from Chinese medicine as “chenpi”, is especially rich in flavonoids and carotenoids, which are believed to confer a variety of health benefits. The use of MOP dates back to ancient times, boasting a longer history than many other flavonoid-based health foods [7]. This peel contains a higher concentration of “hesperidin,” a specific flavonoid, compared to many other agricultural products, making it particularly noteworthy [5,8,9]. Recent pharmacological studies in small animal models have demonstrated that hesperidin and nobiletin, compounds abundant in certain fruits, exhibit protective effects against Alzheimer’s disease [10,11].

Companion animals, particularly dogs, play a crucial role in providing emotional support to their owners, a function with evolutionary roots that may be partially mediated by oxytocin [12]. However, the COVID-19 pandemic has adversely impacted the care and relationships between companion animals and their owners in various ways [13]. For example, a marked increase in requests for vocal cord surgery in senior dogs has been observed, likely attributable to increased barking associated with prolonged indoor confinement during lockdown. These surgical procedures necessitate careful consideration due to their ethical implications and potential impact on animal welfare [14].

In Japan, domestic production of mandarin oranges has been declining annually due to the aging population of mandarin farmers and other contributing factors. According to the Ministry of Agriculture, Forestry, and Fisheries, production in 2022 was less than one-fifth of the peak output in 1975, which was 3.665 million tons, plummeting to 682,000 tons. Early-season satsuma mandarins account for approximately 60% (402,000 tons) of domestic mandarin production; however, roughly 10% is lost due to discrepancies between harvest and shipment volumes [15,16]. Furthermore, the overall discard rate of mandarins, encompassing the proportion of inedible parts such as peels, is approximately 20% [17]. Considering that fruits undergo thinning before harvesting, additional unused resources are expected. Therefore, maximizing the high-value utilization of mandarin peels not only reduces food waste but also enhances the income of mandarin farmers, thereby supporting Japanese agriculture.

Wakayama Prefecture, Japan’s largest mandarin-producing region, benefits from a warm climate ideal for cultivating early-season satsuma mandarins. While these mandarins are frequently consumed fresh, those that do not meet retail standards are processed into juices or canned products, resulting in the disposal of a significant portion of peels and non-standard fruits. Reportedly, hesperidin content in satsuma mandarins is 1.46% in ripe mandarins harvested in December; in contrast, mandarins picked in July exhibit hesperidin contents of 4.98–5.86%, i.e., 3.4–4.0 times higher [18].

This study was undertaken to investigate the presence of beneficial flavonoids in MOP powder, taking into account the current production scenario in Japan, and its applications in dogs. We initiated our investigation by powdering the peels of satsuma mandarins, produced in large volumes domestically, and subsequently analyzed the levels of hesperidin, nobiletin, and various metabolic components in the blood following the administration of MOP to dogs, for which standard samples of other metabolic compounds (Hesperetin-7-O-β-D-glucuronide, Hesperetin-3′-O-beta-D-glucuronide, Homoeriodictyol, Hesperetin) were available. Utilizing the peel powder derived from early-maturing satsuma mandarins (Wakayama Satsuma mandarin: October), rich in both hesperidin and nobiletin—which have recently been identified as effective flavonoids against Alzheimer’s disease in mice models [11,12]—we conducted pharmacokinetic studies in experimental dogs. Additionally, we performed metagenomic analysis of the gut microbiome in the dogs, as alterations in gut microbiota have been associated with cognitive disorders in both humans and animals [19,20,21]. Finally, a pilot study was conducted to evaluate the efficacy of these compounds in three dogs diagnosed with the disease.

## 2. Materials and Methods

### 2.1. Preparation and Component Analysis of Mandarin Orange Peel Powder

#### 2.1.1. Preparation of Mandarin Orange Peel Powder

Dried MOP chips (Kishu Foods Co., Ltd., Wakayama, Japan) were utilized as samples and finely ground using a micro-pulverizer (ACM Pulverizer: Hosokawa Micron Corporation, Osaka, Japan). Thinned mandarin oranges were sourced from farmers in Kinokawa City, Wakayama Prefecture, and Kumamoto Prefecture, Japan. Samples from Kumamoto Prefecture, encompassing both dried and juiced mandarin segments, were prepared for analysis. Approximately 20 kg of dried MOP chips were pulverized into a fine powder, with approximately 500 g of this powder combined with gelatin to create a sample.

#### 2.1.2. Flavonoid Analysis

Hesperidin (purity ≥ 98.5%) and nobiletin (purity ≥ 98%) standards were purchased from Funakoshi Co., Ltd., Tokyo, Japan. Methanol (Kanto Chemical Co., Inc., Tokyo, Japan.) was of liquid chromatography-mass spectroscopy (LC-MS) grade, and dimethyl sulfoxide (FUJIFILM Wako Pure Chemical Corporation, Osaka, Japan.) was of Japanese Industrial Standards (JIS) grade.

Component analysis of MOP powder was performed using high-performance liquid chromatography (HPLC) to measure hesperidin and nobiletin [22], gas chromatography-mass spectrometry (GC-MS) to quantify limonene, and vacuum drying at 70 °C to determine moisture content.

Hesperidin and nobiletin were analyzed by accurately weighing 100 mg of the powder, which was then ultrasonically extracted using 5 mL of methanol/DMSO (1:1, *v*/*v*) at room temperature for 30 min. After centrifugation at 2500 rpm for 10 min, the supernatant was transferred, and an additional 2 mL of solvent was added to the residual solid for further extraction. The combined extracts were transferred to a 10 mL volumetric flask and brought to a final volume. A 1 μL-aliquot of this solution was filtered, diluted as necessary, and injected into an HPLC column (Agilent 1200 Series, Agilent Technologies, Inc., Santa Clara, CA, USA). The analytical column employed was a C30 column, Develosil RPAQUEOUS-AR-5 (4.6 × 250 mm, 5 μm) (Nomura Chemical Co., Ltd., Aichi, Japn.). The mobile phase comprised a 10 mM phosphoric acid aqueous solution and methanol at a flow rate of 1.0 mL/min, commencing with 40% methanol (3 min), followed by a gradient of 40–65% (3–20 min), 65–95% (20–30 min), and held at 95% for an additional 5 min. The injection volume was 10 μL, the column temperature was maintained at 40 °C, and detection was conducted at 285 nm. A methanol/DMSO (1:1, *v*/*v*) solution, prepared in a similar manner without the sample, served as a blank.

Limonene analysis was performed by an external analysis agency, Japan Food Research Laboratories (JFRL), using a foundation-specified method. Limonene analysis necessitates GC-MS; however, owing to the absence of this equipment in our facility, the analysis was outsourced. Although we did not conduct the analysis on cats, which are known to exhibit heightened sensitivity to limonene toxicity, we ensured that the limonene content remained low following processing, as elevated levels may also be unpleasant to dogs. First, 4 g of the sample was combined with 150 mL of water and 8 mL of heptane and distilled for 90 min. The resulting heptane extract (2 mL) was placed in a 20 mL volumetric flask and adjusted to volume with heptane. A 10 μg aliquot of naphthalene-d8 was added as an internal standard to 1 mL of this solution and injected into the GC-MS. GC-MS analysis was performed using a DB-WAX UI (30 m length, 0.25 mm inner diameter, 0.25 μm film thickness, Agilent Technologies, Inc., Santa Clara, CA, USA).) in conjunction with an Agilent 6890N/5975B GC-MS (Agilent Technologies, Inc., Santa Clara, CA, USA)). The initial oven temperature was set at 40 °C for 5 min, then escalated to 110 °C at a rate of 10 °C/min, and subsequently increased to 220 °C at a rate of 15 °C/min. The carrier gas flow rate was maintained at 1.0 mL/min (He). The injector temperature was set to 220 °C, the ion source temperature to 230 °C, and the split ratio was established at 10:1. Electron impact (EI) ionization was employed to acquire the mass spectra. Quantification was executed based on the mass fragment peak area at *m*/*z* 136 (limonene and naphthalene-d8).

### 2.2. Administration Tests on Experimental Dogs

#### 2.2.1. Test Animals

The test animals were four clinically healthy Beagle dogs (*n* = 4, age: 5.5 ± 1.7 years, weight: 9.7 ± 1.7 kg). All experiments were conducted in accordance with Kitasato University guidelines for the care and use of laboratory animals. The study was approved by the Kitasato University Animal Experimentation Ethics Committee (approval numbers: 22-004, 23-059). The dogs’ health status was verified through a comprehensive examination. Initially, prior to the time-course blood concentration study, blood and fecal samples were collected to establish baseline values. Beagle dogs were then administered a single oral dose of 4 g of the compound, followed by peripheral blood collection at 4, 6, and 8 h post-ingestion. Subsequently, after a one-day washout period, intestinal microbiota analysis and blood biochemical tests were conducted using the same dogs. In this phase, Beagle dogs received 1 g of the compound bi-daily for 14 days, after which blood and feces were collected. The collected blood underwent centrifugation at 3000 rpm for 5 min to isolate plasma, while the feces were stored directly in containers. Both samples were frozen and maintained at −80 °C.

#### 2.2.2. Cube Preparation Method

Four hundred fifty grams of commercially available chicken skin (frozen) were heated with 250 mL of tap water. After being heated over medium heat for forty-five minutes, 10 mL of chicken skin broth, 1 g of mandarin peel powder (Wakayama Prefecture, early season mandarin, October), and 0.417 g of gelatin powder were mixed per cube, after which the mixture was allowed to stand at room temperature and then frozen. A blank sample was prepared similarly but without mandarin peel powder.

For the administration test involving the Beagle dogs, finely pulverized dried chips derived from early-season Satsuma mandarins sourced from Wakayama Prefecture in October were utilized. These chips met the ingredient standards of the domestic “Act on Safety Assurance and Quality Improvement of Feeds” (Pet Food Safety Act).

#### 2.2.3. Administration and Sampling

Pre-administration blood and feces collection: Blood samples were centrifuged at 3000 rpm for 5 min to separate plasma, which was then stored at −80 °C. Fecal samples were placed in storage containers and similarly frozen at −80 °C. The collected samples were preserved until analysis for approximately one month under consistent conditions (−80 °C).

Blood collection for the time–course blood concentration study: Four cubes (4 g/head) were orally administered to each dog. Blood was collected at 0, 4, 6, and 8 h post-administration. Fecal and blood samples were obtained for intestinal microbiota analysis and blood biochemical tests, after which the dogs were orally administered the compound twice daily (1 g/head) for two weeks, upon completion of which feces and blood were collected.

### 2.3. Plasma Metabolite Analysis

One hundred microliters of plasma were added to an Eppendorf tube containing 300 μL of acetonitrile/methanol (5:1). After vortexing, the mixture was sonicated for one minute and centrifuged at 14,000 rpm for 10 min. The resultant supernatant was employed as the sample solution. A portion of the sample solution was injected into a Q-TOF LC-MS system (Agilent 6546, Agilent Technologies, Inc., Santa Clara, CA, USA). The analysis column utilized was a C18 column, Unison UK-C18 UP (2.0 × 100 mm, 3 μm) (Intact Corporation., Toronto, Ontario, Canada). The mobile phase consisted of a 0.1% formic acid aqueous solution and acetonitrile at a flow rate of 0.4 mL/min, commencing with 10% acetonitrile (3 min), followed by a gradient of 10–100% (3–20 min), and maintained for 10 min. The injection volume was 1 μL, the column temperature was set to 40 °C, and electrospray ionization (ESI) was employed.

### 2.4. Intestinal Microbiota Analysis

#### 2.4.1. DNA Extraction from Feces

Each 100 mg fecal sample collected before and following the two-week cube intake was suspended in a 4M guanidinium thiocyanate buffer. The suspension underwent processing with a cell disruptor, and the DNA was purified utilizing a GenePrep Star PI-480 automatic DNA isolation system (Kurabo Industries Ltd., Osaka, Japan).

#### 2.4.2. Microbiota Analysis

Using dual-index primers [23] targeting both bacteria and archaea, 16S rDNA was amplified from each fecal DNA sample. Nucleotide sequences were generated as FASTQ files on the MiSeq system (Illumina, San Diego, CA, USA). The FASTQ files underwent analysis using QIIME2 version 2023.5 [24], employing the SILVA database version 138 for taxonomic assignment. From these results, the α-diversity index (Chao1) and β-diversity principal coordinate analysis, including weighted and unweighted Unifrac distances, were computed. Taxonomic analysis was conducted, and the Level 7 classification ratio was further evaluated utilizing linear discriminant analysis Effect Size (LEFSe) to identify bacterial taxa that were significantly altered following administration of MOP [25]. Additionally, PICRUSt2 version 2021.2 [26] and ggpicrust2 version 1.7.3 [27] were employed to predict metabolic changes induced by shifts in microbiota post-administration, referencing the Kyoto Encyclopedia of Genes and Genomes (KEGG) database. Statistical analysis was executed using R version 4.3.2. A paired *t*-test or Wilcoxon signed-rank sum test was employed to assess the significance of changes before and after administration, with a significance threshold of *p* < 0.05. Multiple testing was corrected using the false discovery rate (FDR) < 0.1.

### 2.5. General Condition and Blood Biochemical Tests of Experimental Dogs

The general conditions (appetite, water intake, vitality, defecation and urination, vomiting, and body weight) of the four Beagle dogs were monitored throughout the testing period. Blood biochemical assessments were performed on heparin plasma prior to administration and in the second week following administration, utilizing an automatic biochemical analyzer (Siemens Dimension RxL MAX, Siemens Japan K.K., Tokyo, Japan), quantifying total protein (TP), albumin (ALB), albumin/globulin ratio (A/G), glucose (GLU), total cholesterol (CHO), aspartate aminotransferase (AST), alanine aminotransferase (ALT), alkaline phosphatase (ALP), gamma-glutamyl transpeptidase (GGT), total bilirubin (TB), lactate dehydrogenase (LDH), creatine kinase (CK), blood urea nitrogen (BUN), creatinine (CRE), phosphorus (IP), calcium (CA), bicarbonate (HCO3), sodium (Na), potassium (K), chloride (Cl), anion gap (AG), and the urea nitrogen/creatinine ratio (UN/Cre).

### 2.6. Demonstration Test by Volunteers

With the consent of owners, the palatability of “MOP” as a food supplement was evaluated in Shiba Inu dogs (*n* = 3). One gram of the previously analyzed powder was mixed with a gelling medium (pig collagen, chicken skin collagen, and/or cooking gelatin) at approximately 50 °C and solidified into 1 cm × 1 cm × 1 cm cubes. The resulting solid cubes were administered to a 1-year-old male Shiba Inu, a 3-year-old female Shiba Inu, and a 14-year-old female Shiba Inu, all of which were confirmed to consume the cubes.

Additionally, the owners provided solid cubes orally twice daily to senior companion dogs exhibiting delirium and excessive barking behavior (14-year-old female Shiba Inu, 14-year-old male miniature dachshund, and 19-year-old male miniature dachshund). The volunteer selected a senior dog whose owner expressed concern owing to the dog’s howling, which appeared to be primarily a response to anxiety.

## 3. Results

### 3.1. Component Analysis of Mandarin Orange Peel Powder

The composition of the MOP powder was as follows: hesperidin, 9.3 g/100 g; nobiletin: 41 mg/100 g; limonene: 14 mg/100 g; and moisture, 3.2 g/100 g. Early-season mandarins exhibit a high concentration of hesperidin, and this analysis revealed higher hesperidin levels during the October harvest compared to the December harvest.

Furthermore, an analysis was conducted on whole, thinned mandarins that were dried and powdered instead of solely focusing on the peels. The findings indicated that these whole mandarins also possessed high flavonoid content, suggesting that they could be utilized equivalently to early-season mandarin peels (Table 1, Supplementary Figures S1 and S2).

### 3.2. Administration Tests on Experimental Dogs

#### 3.2.1. Plasma Metabolite Analysis

Plasma metabolite analysis was performed at 0, 4, 6, and 8 h following administration. Hesperidin was detected at concentrations ranging from approximately 5–20 ng/mL in samples taken at 4, 6, and 8 h, with the highest concentration observed at 4 h. However, metabolites such as hesperidin glucuronide and nobiletin-related substances were not detected in any sample.

#### 3.2.2. Intestinal Microbiota Analysis

The 16S rDNA sequence analysis of the V3–4 region of fecal DNA from four dogs, conducted before and after 2 weeks of intake, detected an average of 25,504 ± 3169.5 microbiome features per sample. Statistical analysis was performed to evaluate changes in the microbiota before and after MOP intake.

**Figure 1:**
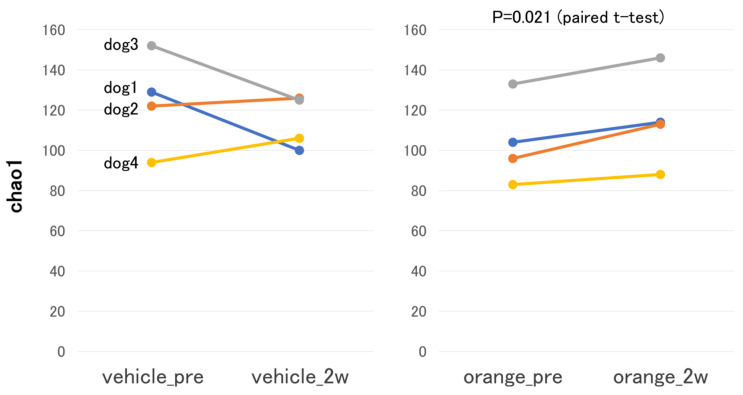
**α-Diversity analysis.** Changes in the chao1 index before (vehicle_pre) and two weeks after (vehicle_2w) administration of cubes without mandarin orange peel (**left**), and changes before (orange_pre) and two weeks after (orange_2w) administration of cubes containing mandarin orange peel (**right**).

No discernible trend was observed in α-diversity changes prior to (vehicle_pre) and two weeks after (vehicle_2w) administration of cubes without MOP. However, when comparing the Chao1 index before (orange_pre) and two weeks after (orange_2w) administration of cubes containing MOP, the Chao1 index increased in all animals at 2 weeks, indicating higher α-diversity (Figure 1). These data were subjected to the Shapiro–Wilk normality testing, which did not reject the assumption of normality (*p*-values: vehicle_pre = 0.539, vehicle_2w = 0.3457, orange_pre = 0.6946, orange_2w = 0.6875). Parametric analysis using a paired *t*-test demonstrated no significant changes in the vehicle group (*p* = 0.41), but significant changes were observed in the orange group (*p* = 0.021). The results of principal coordinate analysis (PCoA) did not reveal clear cluster formation before and after the administration of feed containing MOP. Although the β-diversity distance increased after administration, no significant differences were noted.

**Figure 2:**
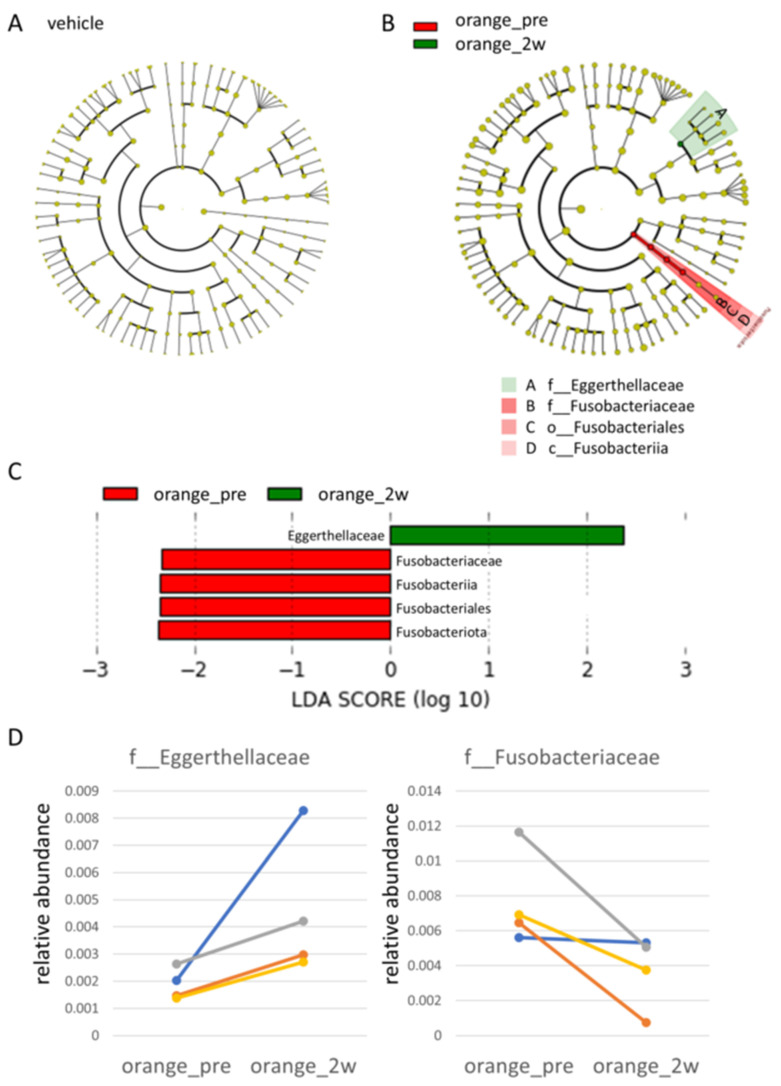
**LEfSe Analysis.** Cladogram of individuals as subclass variables before and after administration of cubes without mandarin orange peel (vehicle_pre, 2w) (**A**), before and after administration of cubes containing mandarin orange peel (orange_pre, 2w) (**B**), and LDA histogram (**C**). The pairwise Wilcoxon test indicates which bacterial species showed significant increases before and after administration, marked in green or red. Additionally, pairwise plots show the relative abundance of *Eggerthellaceae* and *Fusobacteriaceae,* which significantly changed before and after administration of cubes containing mandarin orange peel (**D**). LEfSe, Linear Discriminant Analysis Effect Size.

In the taxonomy analysis, the stacked bar graph of species-level (Level_7) bacterial species average values suggested potential changes before and after administration of the cubes, irrespective of whether they contained MOP. However, neither the Wilcoxon signed-rank sum test nor the paired *t*-test, following multiple testing correction, demonstrated statistically significant changes in bacterial species. Nevertheless, LEfSe analysis with individuals as subclass variables detected a decrease in the *Fusobacteriaceae* family (logLDA = 2.337, *p* = 0.021) and an increase in the *Eggerthellaceae* family (logLDA = 2.37, *p* = 0.021) following MOP administration (Figure 2B,C). These changes were consistently observed across all individuals analyzed (Figure 2D). In microbiota metabolic prediction analysis conducted via PICRUSt, although 14 differences with *p* < 0.05 were identified before and after MOP administration using paired *t*-tests, all significance was negated after multiple testing correction. Furthermore, ggPICrust analysis did not identify any significantly enriched Kyoto Encyclopedia of Genes and Genomes (KEGG) pathways.

### 3.3. General Condition and Blood Biochemical Tests of Experimental Dogs

Throughout the test period, none of the dogs exhibited gastrointestinal symptoms such as diarrhea or vomiting, and no significant changes were detected in appetite, water intake, or body weight. Blood biochemical tests indicated a declining trend in blood urea nitrogen (BUN) across all individuals; however, no significant changes were noted in other parameters (Table 2).

### 3.4. Demonstration Test on Senior Companion Dogs (3 Cases)

**Figure 3:**
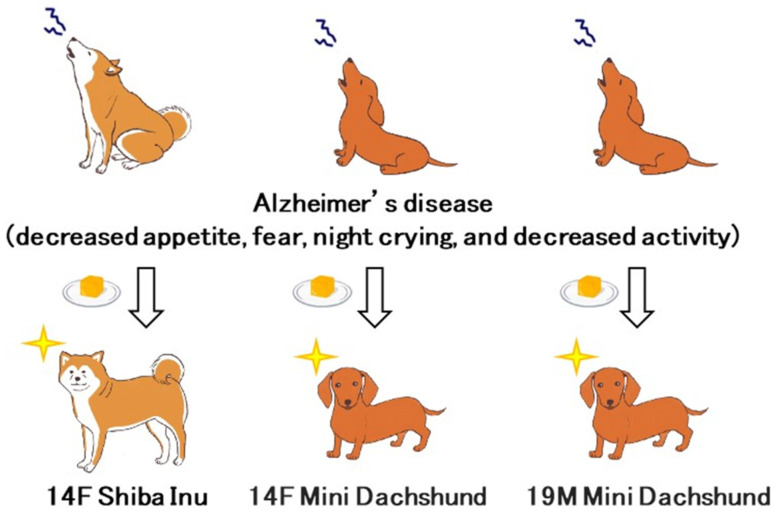
**Demonstration test on senior dogs**. Three senior dogs exhibiting symptoms of Alzheimer’s disease (such as loss of appetite, fear, nighttime crying, and decreased activity) were administered gelatin cubes containing mandarin orange peel (MOP). All three dogs showed improvement in their symptoms.

A 14-year-old female Shiba Inu diagnosed with Alzheimer’s disease (characterized by decreased appetite, fearfulness, night crying, and reduced activity) was administered 2–4 cubes per day for one month (Phase 1). Improvement in all four symptoms was observed from the seventh day of administration, with the dog exhibiting less fear and approaching its owner. Enhanced walking speed was also noted. No side effects or alterations in stool characteristics were documented. After approximately one month, administration was halted, leading to a gradual reappearance of symptoms such as trembling, fear, and circling nine days later (Figure 3). Consequently, the administration of solid cubes was resumed (Phase 2). Unlike Phase 1, the solid cubes were prepared with tuna juice or cooking gelatin to enhance palatability as dog food. Improvement in Parkinsonian-like symptoms was observed again by the seventh day after resuming administration.

Similarly, a 14-year-old female Miniature Dachshund demonstrated reduced barking and dementia symptoms after administration, maintaining good condition nine months following the initiation of treatment. A 19-year-old male Miniature Dachshund exhibited reduced barking and fear post-administration; despite weight loss from 9 kg to 6 kg, he continued to maintain a good appetite nine months after the onset of treatment.

## 4. Discussion

This study analyzed the components present in domestic mandarin peel. The results indicated that, per 100 g of dried powder, hesperidin was found at a concentration of 9.3 g, and nobiletin was present at 41 mg. Notably, early-harvested mandarins from Wakayama exhibited higher concentrations of hesperidin, with October harvests yielding greater concentrations than the December harvests. This observation suggests that early harvesting could be a beneficial strategy to stabilize agricultural work throughout the year in periods of anticipated overproduction.

Recent pharmacological studies employing small animal models have demonstrated that hesperidin and nobiletin—compounds present in certain fruits—exhibit effectiveness against Alzheimer-like diseases [11,12]. In a mouse model, hesperidin demonstrated the capacity to reduce elevated levels of phosphorylated tau (p-Tau) protein [11], while nobiletin significantly decreased the accumulation of β-amyloid in both the hippocampus and cortex, indicating potential cognitive function improvements in these mice [12]. The noted improvements in the demonstration test with senior dogs are likely attributable to the intake of MOP, rich in these two flavonoids that operate through distinct pathways. Additionally, studies have suggested that mandarin peels, which contain hesperidin and nobiletin, may aid in alleviating dementia symptoms [28,29,30]. Notably, hesperidin has been shown to exert anti-inflammatory effects in the brain at the test tube level, and clinical trials have reported that a dietary supplement rich in hesperidin enhances cerebral blood flow, cognition, and memory in human subjects. This notion supports the hypothesis that hesperidin may have contributed to alleviating the abnormal behavior in dogs suffering from Alzheimer’s disease. Meanwhile, the results from the intestinal microbiota analysis reveal that the administration of samples containing MOP increased the α-diversity of the microbiota. A decrease in intestinal microbiota diversity is commonly observed in patients with Alzheimer’s disease, alongside conditions such as obesity and metabolic syndrome [31], suggesting that any recovery in diversity could positively influence central nervous system functioning [19]. Taxonomic analysis revealed a decrease in the *Fusobacteriaceae* family and an increase in the *Eggerthellaceae* family following MOP administration. The phylum Fusobacteria is regarded as a pathogenic bacterium that tends to increase with age, contributing to inflammation and DNA damage, and is suspected to have associations with colorectal cancer [32], though there are no established links to dementia. Conversely, the *Eggerthellaceae* family is recognized for its increase with age and association with age-related health issues such as frailty, metabolic abnormalities, and chronic inflammation, thereby potentially increasing the risk of cognitive decline [20]. However, existing literature suggests correlations with the integrity of white matter regions involved in language function, memory, and learning ability [33]. Bacteria within the *Eggerthellaceae* family produce plant-derived bioactive compounds, particularly equol, which possesses estrogenic properties similar to soy isoflavones [34]. Equol has demonstrated the ability to inhibit arteriosclerosis and cognitive decline. Although the sample size in this study was limited, the observed improvements in symptoms of Alzheimer’s disease in senior dogs suggest that components from MOP may have potential in ameliorating dementia-like conditions. Nonetheless, PICRUSt metabolic predictions did not reveal significant changes in any pathway following multiple testing corrections, indicating that further investigation is necessary to ascertain whether MOP contributes to the generation of plant-derived bioactive compounds alongside intestinal bacteria. Hesperidin is swiftly metabolized upon oral administration, leading to the formation of hesperetin–glucuronide and homoeriodictyol–glucuronide conjugates [35]. Furthermore, sugar-modified forms of hesperidin have been developed to enhance absorption [36]. In this study, the use of mandarin peel powder encapsulated in gelatin cubes facilitated ingestion, which may have culminated in favorable impacts on the microbiome.

Among companion animals, aging in dogs has emerged as a particularly pressing issue [37]. In recent years, especially during the COVID-19 pandemic, restrictions on the outdoor activities of senior owners, alongside other factors, have precipitated significant mental health challenges for both pets and their owners [13,38]. The clinical pilot trial conducted as part of this study was executed under severely constrained conditions amid Japan’s COVID-19 crisis. With the owner’s consent, three senior dogs presenting symptoms indicative of Alzheimer’s were administered dried mandarin peel powder, incorporated into collagen cubes. Initially, a 14-year-old female Shiba Inu, exhibiting severe loss of appetite, fear, night crying, and reduced activity, was given 2 to 4 cubes each day for one month. Commencing on the seventh day of treatment, all four symptoms exhibited improvement. Additionally, a cessation trial was conducted; after discontinuation of treatment, nine days later, the dog gradually exhibited symptoms such as trembling, fear, and clockwise circling. However, upon resuming administration, these Parkinsonian-like symptoms also improved.

Additionally, cooperation was acquired from the owner of a Miniature Dachshund displaying pronounced Alzheimer’s-like symptoms. Both a 14-year-old female and a 19-year-old male dog received the identical dosage of the supplement. Throughout the observation period, no side effects, such as alterations in stool consistency or other adverse symptoms, were noted. Three senior dogs exhibiting howling symptoms were included in the study, and their owners were compelled to maintain them indoors due to the COVID-19 crisis. Although efficacy tests are typically conducted as double-blind trials with a placebo control, this study was primarily concentrated on pharmacological assessments involving experimental dogs (Beagles) and an analysis of alterations in intestinal microbiota. Future efforts will aim to include senior companion dogs exhibiting symptoms of Alzheimer’s disease in conjunction with veterinary clinical trials utilizing a placebo control.

While proper drug efficacy trials in humans ideally should be conducted under double-blind conditions, testing with senior subjects manifesting Alzheimer’s disease presents significant challenges. Additionally, the safety of fruit-derived flavonoids, such as dried mandarin peels utilized as health supplements, indicates that the long-term benefits of such supplementation merit further investigation in forthcoming studies [39,40].

## 5. Conclusions

Herein, we analyzed the flavonoids (hesperidin and nobiletin) present in the peels of mandarin oranges from Japan’s major producing regions. The early-ripening varieties (harvested in October) from Wakayama exhibited high concentrations of these compounds. Although plasma metabolite analysis indicated limited absorption of these compounds following oral administration in experimental dogs, the gut microbiota analysis revealed significant changes in the intestinal composition. Notably, there was a marked decrease in *Fusobacteriaceae* and an increase in *Eggerthellaceae* after MOP intake, suggesting potential prebiotic effects. Based on prior reports indicating the efficacy of both flavonoids in improving symptoms in Alzheimer’s disease in rodent models, we initially conducted pharmacokinetic studies in experimental dogs and evaluated anti-Alzheimer’s effects through intestinal microbiota analysis. Behavioral assessments indicated that the three tested dogs exhibited reductions in anxiety-related behaviors and improvement in overall activity levels, with the benefits observable within a week of MOP administration. Importantly, no adverse gastrointestinal effects were noted, indicating the safety of MOP as a dietary supplement for canine use. These preliminary results support the potential application of MOP as a natural intervention for enhancing gut health and cognitive function in senior dogs. Additional research is recommended to further elucidate the mechanistic pathways underlying these observed benefits.

## Figures and Tables

**Table 1:** Component Analysis Results of Various Powder Samples.

Powder Sample	Hesperidin	Nobiletin	Limonene	Moisture
Peel of Early-Ripening Mandarin (Wakayama Satsuma Mandarin: Harvested in October 2021)	9.3 g	41 mg	14 mg	3.2 g
Peel of Mandarin (Domestic Satsuma Mandarin: Harvested in December 2019)	6.9 g	35 mg	20 mg	4.5 g
Powdered Peel of Arita Mandarin (Commercial Product)	6.4 g *	32 mg *	23 mg *	N/A
Thinned Mandarin (Kinokawa City, Wakayama Prefecture: Harvested in August 2024)	7.4 g	21 mg	75 mg	1.9 g
Thinned Mandarin, Dried After Cutting (Kumamoto Prefecture: Harvested in July 2023)	14 g	44 mg	310 mg	2.7 g
Thinned Mandarin, Dried After Juicing (Kumamoto Prefecture: Harvested in July 2023)	14 g	41 mg	46 mg	2.8 g

* Values are quoted from the product display. Unit: per 100 g.

**Table 2:** Blood Biochemical Test Results.

**Parameter**		**TP**	**ALB**	**A/G** **Ratio**	**GLU**	**CHO**	**AST**	**ALT**	**ALP**	**GGT**	**TB**	**LDH**
**Unit**		**g/dL**	**g/dL**	**-**	**mg/dL**	**mg/dL**	**IU/L**	**IU/L**	**IU/L**	**IU/L**	**mg/dL**	**IU/L**
Pre	1	6.6	3.0	0.83	107	155	31	45	29	4.0	0.04	37
	2	6.7	3.2	0.97	109	232	28	31	58	1.0	0.05	12
	3	6.2	2.8	0.82	99	200	36	37	50	5.3	0.02	46
	4	6.1	3.3	1.18	107	164	29	111	25	4.1	0.02	18
	Mean ± SD	6.4 ± 0.3	3.1 ± 0.2	0.95 ± 0.17	106 ± 4	188 ± 35	31 ± 4	56 ± 37	41 ± 16	3.6 ± 1.8	0.03 ± 0.02	28 ± 16
2w	1	6.3	3.1	0.97	104	159	30	41	37	10	0.10	71
	2	6.4	3.1	0.94	102	210	30	40	41	10	0.10	86
	3	6.3	3.2	1.03	93	224	28	34	52	10	0.10	96
	4	6.0	3.3	1.22	98	175	35	69	33	10	0.10	64
	Mean ± SD	6.3 ± 0.2	3.2 ± 0.1	1.04 ± 0.13	99 ± 5	192 ± 30	31 ± 3	46 ± 16	41 ± 8	10 ± 0	0.10 ± 0.00	79 ± 14
**Parameter**		**CK**	**BUN**	**CRE**	**IP**	**CA**	**HCO3**	**Na**	**K**	**Cl**	**AG**	**UN/Cre** **Ratio**
**Unit**		**IU/L**	**mg/dL**	**mg/dL**	**mg/dL**	**mg/dL**	**mmol/L**	**mEq/L**	**mEq/L**	**mEq/L**	**mEq/L**	**-**
Pre	1	97	18	0.54	5.1	9.8	21	150	4.2	111	22	33
	2	72	22	0.43	5.4	10.1	21	149	4.3	110	22	50
	3	282	28	0.41	3.5	9.5	24	146	4.3	106	20	69
	4	77	19	0.58	3.7	9.9	21	149	4.3	112	21	32
	Mean ± SD	132 ± 101	22 ± 5	0.49 ± 0.08	4.4 ± 1.0	9.8 ± 0.3	22 ± 2	149 ± 2	4.3 ± 0.0	110 ± 3	21 ± 1	46 ± 17
2w	1	46	10	0.64	4.0	9.9	14	143	4.2	119	15	16
	2	51	9.1	0.54	3.8	9.8	14	141	4.3	120	12	17
	3	62	8.9	0.49	2.7	9.6	10	141	3.8	122	13	18
	4	38	6.3	0.69	2.6	9.7	13	142	3.7	120	12	9
	Mean ± SD	49 ± 10	8.6 ± 1.6	0.59 ± 0.09	3.3 ± 0.7	9.8 ± 0.1	13 ± 2	142 ± 1	4.0 ± 0.3	120 ± 1	13 ± 1	15 ± 4

pre: before administration. 2w: 2 weeks after administration

## Data Availability

The data presented in this study are available on request from the corresponding author, E.K., due to the inclusion of analysis data from external institutions.

## Supplementary Materials

The following supporting information can be downloaded at: https://www.mdpi.com/article/10.3390/metabo15010003/s1, Figure S1: HPLC Chromatogram of Hesperidin and Nobiletin, Figure S2: GC/MS Chromatogram of Limonen.
