# Supplementary material for: Exploring the Pharmacokinetics and Gut Microbiota Modulation of Hesperidin and Nobiletin from Mandarin Orange Peel in Experimental Dogs: A Pilot Study
Source: Metabolites. 2024 Dec 25;15(1):3. doi: 10.3390/metabo15010003 (PMC11766869; doi:10.3390/metabo15010003)
Supplement: Supplementary file 1 [file metabolites-15-00003-s001.zip › metabolites-3362040-supplementary.pdf]

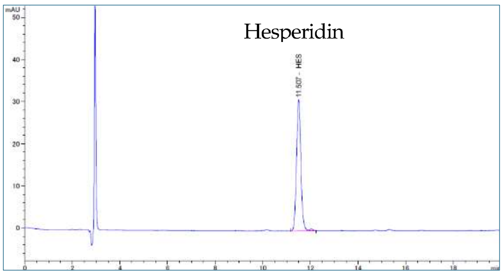

Standard Solution (Hesperidin)

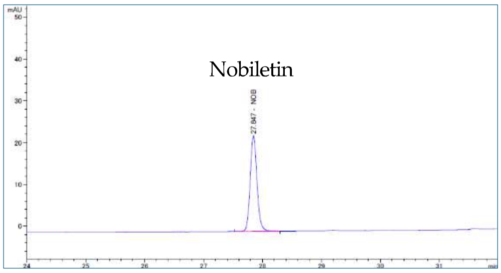

Standard Solution (Nobiletin)

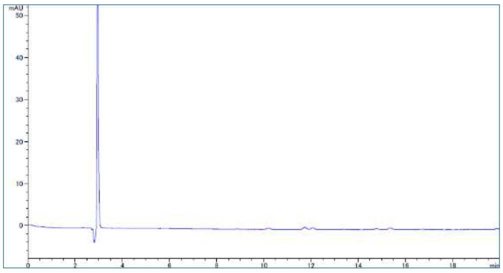

Blank Solution

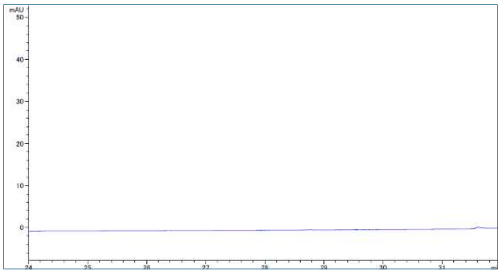

Blank Solution

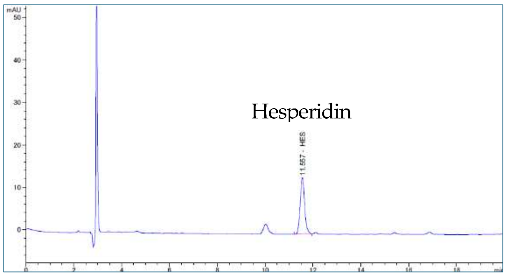

100x Diluted Sample Solution: Peel of Early-Ripening Mandarin

(Wakayama Satsuma Mandarin: Harvested in October 2021)

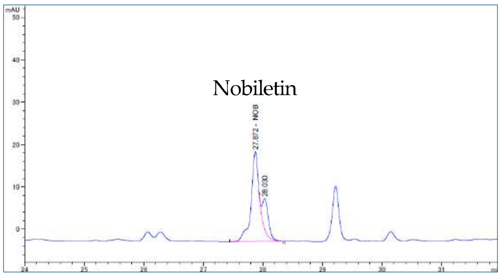

Sample Solution: Peel of Early-Ripening Mandarin

(Wakayama Satsuma Mandarin: Harvested in October 2021)

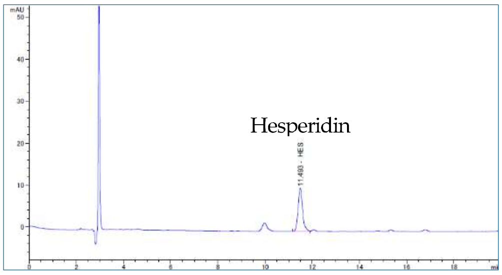

100x Diluted Sample Solution: Peel of Mandarin

(Domestic Satsuma Mandarin: Harvested in December 2019)

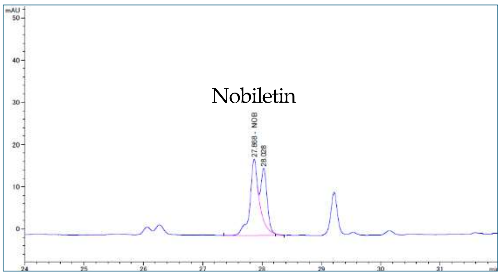

Sample Solution: Peel of Mandarin

(Domestic Satsuma Mandarin: Harvested in December 2019)

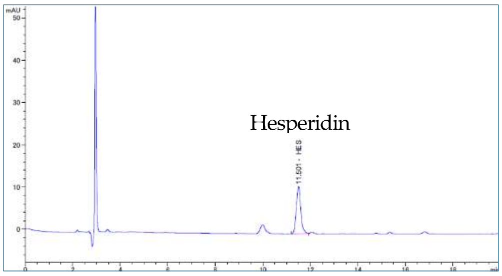

100x Diluted Sample Solution: Thinned Mandarin

(Kinokawa City, Wakayama Prefecture: Harvested in August 2024)

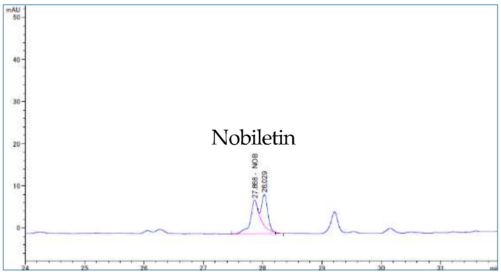

Sample Solution: Thinned Mandarin

(Kinokawa City, Wakayama Prefecture: Harvested in August 2024)

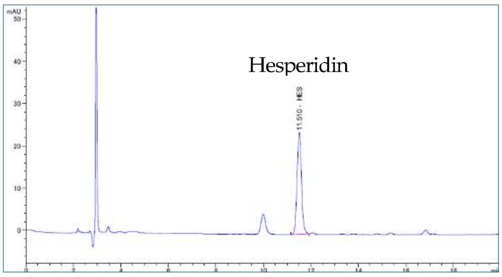

100x Diluted Sample Solution: Thinned Mandarin, Dried After Juicing

(Kumamoto Prefecture: Harvested in July 2023)

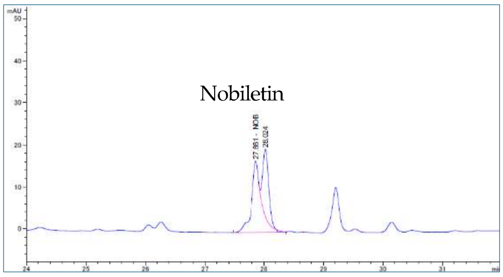

Sample Solution: Thinned Mandarin, Dried After Juicing

(Kumamoto Prefecture: Harvested in July 2023)

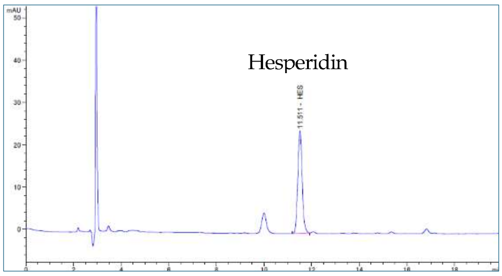

100x Diluted Sample Solution: Thinned Mandarin, Dried After Cutting

(Kumamoto Prefecture: Harvested in July 2023)

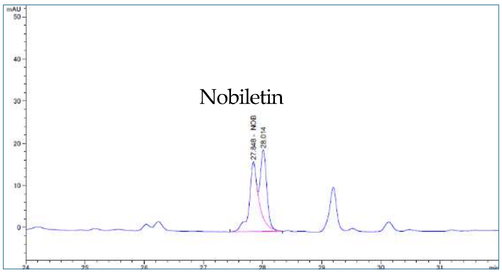

Sample Solution: Thinned Mandarin, Dried After Juicing

(Kumamoto Prefecture: Harvested in July 2023)

Figure S1. HPLC Chromatogram

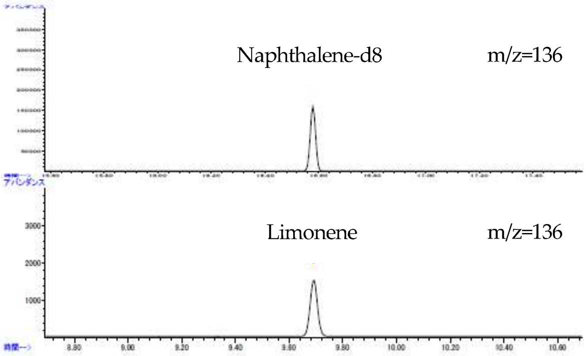

Standard Solution (Limonene)

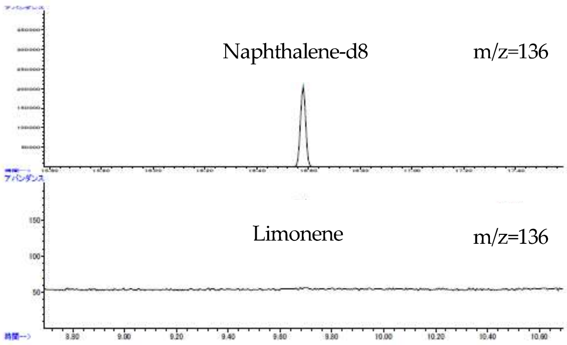

Blank Solution

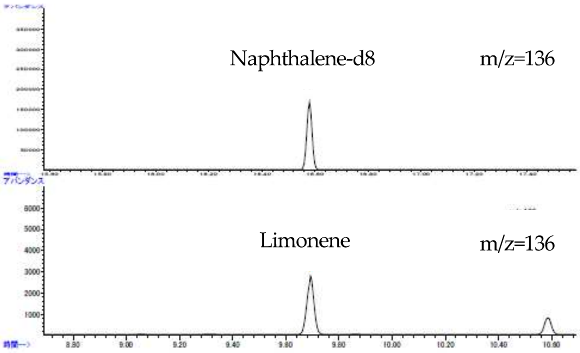

Sample Solution : Thinned Mandarin

(Kinokawa City, Wakayama Prefecture: Harvested in August 2024)

Figure S2. GC/MS Chromatogram
